# Vaccination status and long COVID symptoms in patients discharged from hospital

**DOI:** 10.1038/s41598-023-28839-y

**Published:** 2023-02-11

**Authors:** Teresa Cristina D. C. Nascimento, Livia do Valle Costa, Amanda Danieletto Ruiz, Carla B. Ledo, Valeria Paes Lima Fernandes, Luiz Francisco Cardoso, José Mauro Vieira Junior, Roberta Saretta, Roberto Kalil-Filho, Luciano F. Drager

**Affiliations:** 1 Hospital Sírio Libanês, São Paulo, Brazil; 2 Hospital Sírio Libanês, Brasília, Brazil; 3 Instituto do Coração (InCor), Hospital das Clínicas da Faculdade de Medicina da Universidade de São Paulo, São Paulo, Brazil

**Keywords:** Health care, Signs and symptoms, Infectious diseases

## Abstract

Effective vaccination against coronavirus mitigates the risk of hospitalisation and mortality; however, it is unclear whether vaccination status influences long COVID symptoms in patients who require hospitalisation. The available evidence is limited to outpatients with mild disease. Here, we evaluated 412 patients (age: 60 ± 16 years, 65% males) consecutively admitted to two Hospitals in Brazil due to confirmed coronavirus disease 2019 (COVID-19). Compared with patients with complete vaccination (n = 185) before infection or hospitalisation, those with no or incomplete vaccination (n = 227) were younger and had a lower frequency of several comorbidities. Data during hospitalisation revealed that the no or incomplete vaccination group required more admissions to the intensive care unit (ICU), used more corticosteroids, and had higher rates of pulmonary embolism or deep venous thrombosis than the complete vaccination group. Ninety days after hospital discharge, patients with no or incomplete vaccination presented a higher frequency of symptoms (≥ 1) than patients with complete vaccination (40 vs. 27%; p = 0.013). After adjusting for confounders, no or incomplete vaccination (odds ratio [OR] 1.819; 95% confidence interval [CI] 1.175–2.815), female sex (OR 2.435; 95% CI 1.575–3.764) and ICU admission during hospitalisation (OR 1.697; 95% CI 1.062–2.712) were independently associated with ≥ 1 symptom 90 days after hospital discharge. In conclusion, even in patients with severe COVID-19, vaccination mitigates the probability of long COVID symptoms.

## Introduction

Coronavirus disease 2019 (COVID-19) promotes a significant burden of symptoms in the subacute and long term post infection^1^. The commonly reported residual symptoms include fatigue, dyspnoea, and chest pain, among others (collectively described as post-acute COVID-19 syndrome or long COVID)^1–4^. From 2021, progressive implementation of effective vaccination against severe acute respiratory syndrome coronavirus 2 (SARS-CoV-2) has mitigated the risk of complications, including hospitalisations and mortality^5,6^. Recent reports suggest that vaccination can reduce long COVID in patients who do not need hospitalisation^7–10^. Therefore, it is unclear whether vaccination status influences the magnitude of long COVID symptoms in patients with more severe disease who frequently require more intensive support and are more susceptible to long-term complications. In the current study, we systematically evaluated several long COVID symptoms 90 days after hospital discharge in consecutive patients, according to vaccination status. We hypothesised that patients with no or incomplete vaccination would present at least one symptom compared to patients with complete vaccination (at least two doses for all vaccines but ≥ 1 dose for the Janssen™ vaccine, as described by others^10^). Moreover, no or incomplete vaccination would be independently associated with persistence of symptoms after hospital discharge.

## Methods

The study was reviewed by an Institutional Review Board (CAAE 45978621.9.0000.5461) and deemed exempt from IRB oversight (Instituto de Ensino e Pesquisa, IEP, Hospital Sírio Libanês). The requirement of informed consent was waived. All methods were performed in accordance with relevant guidelines and regulations. All data were analysed in a secure, anonymised database physically separated from the main production server.

We studied consecutive hospitalised patients at the Hospital Sirio Libanês, Sao Paulo, and Hospital Sirio Libanês, Brasilia, Brazil from May 2021 to February 2022. All patients had a confirmed diagnosis of COVID-19 based on the presence of related symptoms and a positive result on a SARS-CoV-2 polymerase chain reaction assay of both nasal and pharyngeal swab specimens.

All the collected data were reviewed by the study team to ensure accuracy. The registry utilised a web-based case report form in the RedCap™ platform (Nashville, TN, US).

### Variables and clinical outcomes collected at hospital

Participant data were collected until hospital discharge. Vaccination status was checked in the medical records (routinely documented through a standard vaccination card). Complete vaccination was considered when patients received two or more doses for all vaccines but ≥ 1 dose for the Janssen™. Otherwise, it was considered an incomplete vaccination^10^. We compiled data on demographic characteristics, medical history, length of hospital stay, intensive care unit (ICU) admission, mechanical ventilation, pulmonary embolism (PE), deep venous thrombosis (DVT), major bleeding, need for dialysis, and medications used during hospitalisation (including anticoagulants, convalescent plasma, and corticosteroids).

### Variables collected after hospital discharge

The Clinical Outcomes team from Hospital Sirio Libanes prospectively applied a standardised questionnaire 90 days after hospital discharge, contacting all patients over telephone. All members of this team received formal training to identify and characterise symptoms. They had no access to the details of in-hospital admission. We evaluated the presence of symptoms, such as dyspnoea, tiredness or fatigue, cough, chest pain, sore throat, anosmia or ageusia, headache, arthralgia, myalgia, and others (e.g. diarrhoea and vomiting). Long COVID was defined as the continuation or development of new symptoms 3 months after the initial SARS-CoV-2 infection^11^.

### Statistical analysis

Continuous variables were described as mean ± standard deviation or median and interquartile range; categorical variables were described using absolute and relative frequencies. Comparisons of patients according to vaccination status were performed using chi-square tests for categorical variables and Mann–Whitney tests for continuous variables. Multivariate logistic regression analysis for the presence of ≥ 1 symptoms after 90 days of hospital discharge was presented considering variables with biological relevance for the main outcomes. These included vaccination status, age, sex, diabetes, previous cardiovascular disease, chronic kidney disease, ICU admission, PE or DVT, and corticosteroid use (the last three variables during hospitalisation). All analyses were performed using IBM SPSS Statistics version 24 (Statistical Package for the Social Sciences, SPSS Inc., Chicago, IL).

## Results

**Figure 1:**
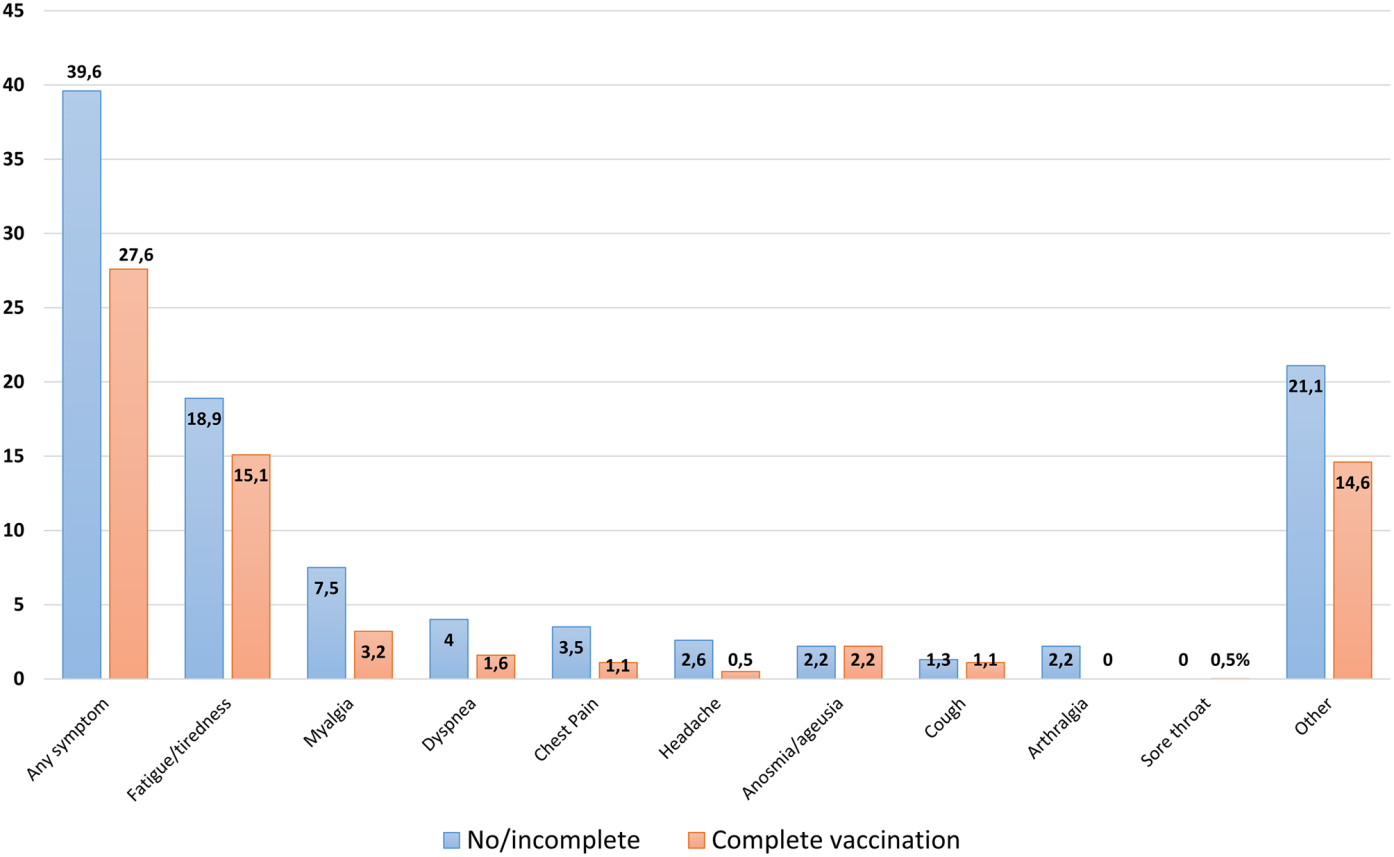
The frequency (**A**) and number of symptoms reported by patients according to vaccination status.

During the study recruitment period, 412 patients were discharged from the hospital and had available data on vaccination status and symptoms after 90 days (227 with no or incomplete vaccination and 185 with complete vaccination). Figure 1 shows the symptoms reported 90 days after discharge. Patients with no or incomplete vaccination had a higher frequency of symptoms (≥ 1) than patients with complete vaccination (p = 0.013). Table 1 shows the main characteristics of the included patients, stratified according to vaccination status. Compared with patients with complete vaccination, those with no or incomplete vaccination were younger and had a lower frequency of hypertension, diabetes, dyslipidaemia, previous cardiovascular disease, and chronic kidney disease. Data during hospitalisation revealed that they required more admissions to the ICU and more corticosteroids and had higher rates of PE or DVT.

**Table 1:** Characteristics of patients discharged after hospitalisation for COVID-19 stratified according to vaccination status.

Characteristics	Total	No or incomplete vaccination	Complete vaccination	p-value
	(n = 412)	(n = 227)	(n = 185)	
Demographic and anthropometric characteristics and comorbidities				
Age (years), median (interquartile range)	60 (48–72)	55 (43–64)	71 (59–78)	** < 0.001**
Male, n (%)	266 (64.6)	156 (68.7)	110 (59.5)	0.051
Self-reported white, n (%)*	277 (85.5)	131 (83.4)	146 (87.4)	0.475
Body mass index (kg/m^2^), median (interquartile range)	28.1 (25.5–31.7)	28.3 (25.6–32.0)	28.0 (25.2–31.1)	0.153
Self-reported previous COVID, n (%)	5 (1.2)	1 (0.4)	4 (2.2)	0.179
Current smoking, n (%)	25 (6.1)	14 (6.2)	11 (5.9)	0.255
Hypertension, n (%)	178 (43.2)	79 (34.8)	99 (53.5)	** < 0.001**
Diabetes, n (%)	90 (21.8)	40 (17.6)	50 (27.0)	**0.022**
Dyslipidaemia, n (%)	93 (22.6)	40 (17.6)	53 (28.6)	**0.008**
Previous cardiovascular disease, n (%)	65 (15.8)	22 (9.7)	43 (23.2)	** < 0.001**
Previous cerebrovascular disease, n (%)	8 (1.9)	4 (1.8)	4 (2.2)	> 0.999
Chronic obstructive pulmonary disease/asthma, n (%)	36 (8.7)	19 (8.4)	17 (9.2)	0.770
Chronic kidney disease, n (%)	13 (3.2)	1 (0.4)	12 (6.5)	** < 0.001**
Self-reported diagnosis of anxiety, n (%)	22 (5.3)	16 (7.0)	6 (3.2)	0.087
Self-reported diagnosis of depression, n (%)	26 (6.3)	12 (5.3)	14 (7.6)	0.344
Data during hospitalisation				
Days of hospitalisation, median (interquartile range)	8.0 (4.0–13.0)	8.0 (4.5–14.0)	8.0 (4.0–13.0)	0.435
Intensive care unit admission, n (%)	107 (26.0)	71 (31.3)	36 (19.5)	**0.007**
Mechanical ventilation, n (%)	43 (10.4)	28 (12.3)	15 (8.1)	0.163
Pulmonary embolism, n (%)	19 (4.6)	15 (6.6)	4 (2.2)	**0.032**
Deep vein thrombosis, n (%)	10 (2.4)	10 (4.4)	0 (0)	**0.003**
Dialysis, n (%)	7 (1.7)	6 (2.6)	1(0.5)	0.135
Major bleeding, n (%)	13 (3.2)	9 (4.0)	4 (2.2)	0.298
Critical illness polyneuropathy, n (%)	9 (2.2)	7 (3.1)	2 (1.1)	0.195
Convalescent plasma, n (%)	10 (2.4)	5 (2.2)	5 (2.7)	0.759
Prophylactic or therapeutic anticoagulants, n (%)*	402 (97.6)	220 (96.9)	182 (98.4)	0.522
Corticoids, n (%)	353 (85.7)	207 (91.2)	146 (78.9)	** < 0.001**

*n = 324 (some patients refused to report this variable).

Bold numbers indicates variables with statistical significance.

After adjusting for multiple confounders, no or incomplete vaccination, female sex, and ICU admission during hospitalisation were independently associated with at least one symptom 90 days after hospital discharge (Table 2).

**Table 2:** Multivariate analysis evaluating the independent variables associated with symptoms 90 days after hospitalization due to COVID-19.

	Coefficient	Odds ratio	95% confidence interval		p value
Group (no or incomplete vaccination)	**0.598**	**1.819**	**1.175**	**2.815**	**0.007**
Age (years)	0.003	1.003	0.987	1.018	0.737
Sex (female)	**0.890**	**2.435**	**1.575**	**3.764**	** < 0.001**
Diabetes (yes)	0.395	1.485	0.890	2.478	0.130
Previous cardiovascular disease (yes)	− 0.167	0.846	0.451	1.589	0.604
Chronic kidney disease (yes)	− 0.813	0.444	0.086	2.292	0.332
Intensive care unit admission during hospitalization (yes)	**0.529**	**1.697**	**1.062**	**2.712**	**0.027**
Pulmonary embolism or deep venous thrombosis during hospitalization (yes)	− 0.634	0.531	0.197	1.427	0.209
Corticoid use during hospitalization (yes)	0.508	1.662	0.840	3.287	0.145
Constant	− 1.481				

Bold numbers indicates variables with statistical significance.

## Discussion

This study evaluated the potential impact of vaccination on long COVID symptoms after hospital discharge and the following results were obtained: despite patients with complete vaccination being older and having a substantially high frequency of several comorbidities (probably because worldwide vaccination strategies initially focused on vulnerable individuals), they presented a lower frequency of symptoms (≥ 1) 90 days after hospital discharge. This result may be partially explained by the fact that patients with no or incomplete vaccination had more complications during hospitalisation than those who received complete vaccination (Table 1). However, multivariate analysis revealed that complete vaccination was independently associated with a lower rate of symptoms (≥ 1) 90 days after hospital discharge. Taken together, these results emphasise the role of vaccination in promoting a lower chance of long COVID after hospital discharge.

The evidence supporting the impact of vaccination on long COVID is scarce and limited to mild cases^7–10,12^. In a survey conducted in a community-dwelling population from the United Kingdom^10^, 23.7% reported long COVID symptoms regardless of severity, at least once during follow-up (at least 12 weeks post infection). The likelihood of long COVID symptoms decreased after vaccination^10^. However, only 3.2% of this population required hospitalisation during the acute phase. A recent systematic review^12^ of six studies (including preprints) revealed that vaccination before SARS-CoV-2 infection could reduce the risk of subsequent long COVID. Overall, the studies reported long COVID in patients who were not hospitalised^12^. Therefore, our study adds to the literature not only by providing consistent results but also by demonstrating that the benefits of vaccination on long COVID are also observed in more severe cases (i.e. patients with SARS-CoV-2 infection who require hospitalisation).

To date, there have been no definitive answer to why there is a lower rate of long COVID after vaccination. Vaccination can increase antibody titres and potentially eliminate viral reservoirs^13^. It is conceivable that abnormalities in T cells, platelets, vascular endothelium, and clotting factors, related to COVID-19^14,15^, can be mitigated by vaccination. Consistent with these previous findings, hospitalised patients who completed the vaccination had a lower rate of significant complications, including thrombotic events.

Our study has some strengths. We included consecutive patients hospitalised due to confirmed COVID-19, from two hospitals. Further, we used standard procedures for checking vaccination status and performing a structured interview looking for symptoms 90 days after hospital discharge, with no access to the vaccination status or the severity of hospitalisation. However, we need to acknowledge the following study limitations: (1) the influence of vaccine type on outcomes (long COVID) could not be tested. There are several potential combinations that that limit our ability to detect any statistically significant differences in long COVID symptoms. (2) At the time of data collection, the third and fourth doses were just implemented in the Brazilian Vaccination Program. Therefore, the number of patients hospitalised despite receiving three or four doses was small. In addition, this finding is explained by the higher protection offered to patients who received extra doses during hospital admissions^16,17^. (3) Another limitation is that the follow-up was short and the 90-day symptoms relied on the patients’ own notification. Multiple evaluations overtime would be interesting to explore the potential impact of complete vaccination on the dynamic changes of symptoms. (4) Finally, we did not explore whether the impact of complete vaccination on long COVID might be influenced by variants of SARS-CoV-2. The lack of genetic confirmation and the relative sample size prevented the utility of this analysis.

In conclusion, our results suggest that effective vaccination may decrease the burden of long COVID in patients who require hospitalisation. Therefore, these findings add to the recent literature suggesting the multiple benefits of vaccination in COVID-19 infection, hospital admissions, severity of hospitalisation, and long COVID.

## Data Availability

The datasets generated and/or analysed during the current study are available from the corresponding author upon reasonable request.
